# Association of High-Risk Obstructive Sleep Apnea with Microvascular Complications in Adults with Type 1 Diabetes Mellitus: A Case–Control Study

**DOI:** 10.3390/jcm15082901

**Published:** 2026-04-10

**Authors:** Selin Cakmak Demir, Adnan Batman, Dilek Yazici, Oguzhan Deyneli, Yüksel Peker

**Affiliations:** 1 Department of Endocrinology, Koc University School of Medicine, 34010 Istanbul, Türkiye; seldemir@kuh.ku.edu.tr (S.C.D.); dilekdy2002@yahoo.com (D.Y.); odeyneli@kuh.ku.edu.tr (O.D.); 2 Department of Endocrinology, Acıbadem University School of Medicine, 34752 Istanbul, Türkiye; dradnan54@hotmail.com; 3 Department of Pulmonary Medicine, Koc University School of Medicine, 34010 Istanbul, Türkiye; 4 Department of Clinical Sciences, Respiratory Medicine and Allergology, Faculty of Medicine, Lund University, 22100 Lund, Sweden; 5 Department of Molecular and Clinical Medicine, Sahlgrenska Academy, University of Gothenburg, 40530 Gothenburg, Sweden; 6 Division of Pulmonary, Allergy, and Critical Care Medicine, University of Pittsburgh School of Medicine, Pittsburgh, PA 15213, USA

**Keywords:** type 1 diabetes mellitus, obstructive sleep apnea, Berlin Questionnaire, microvascular complications, diabetic retinopathy, diabetic neuropathy

## Abstract

**Background**: Obstructive sleep apnea (OSA) is a common but underrecognized sleep-related breathing disorder characterized by intermittent hypoxemia and autonomic dysfunction. OSA prevalence and clinical relevance in Type 1 Diabetes Mellitus (T1DM), particularly in relation to diabetes-related vascular complications, remain insufficiently explored. **Objective**: The aim of this study was to evaluate the prevalence of high-risk OSA in adults with T1DM and controls, and to investigate the association between high-risk OSA and microvascular complications among individuals with T1DM. **Methods**: In this cross-sectional case–control study, 102 adults with T1DM and 126 controls were included. OSA risk was assessed using the modified Berlin Questionnaire (mBQ). Analyses of vascular complications were restricted to participants with T1DM. Multivariable logistic regression models adjusted for age and sex were used to assess associations, with additional adjustments for body mass index, hypertension, current smoking, alcohol use and glycated hemoglobin A1c. **Results**: High-risk OSA was identified in 18.6% of individuals with T1DM and 11.9% of controls, with no significant difference between groups. Among participants with T1DM, the prevalence of microvascular complications (retinopathy and/or neuropathy) was substantially higher in those with high-risk OSA compared with those at low risk (68.4% vs. 18.07%, *p* <0.001). In univariate logistic regression analysis, high-risk OSA was significantly associated with microvascular complications (odds ratio [OR] 4.85; 95% confidence interval [CI] 1.65–14.24; *p* = 0.004). This association remained significant in the fully adjusted model (OR 5.55; 95% CI 1.36–22.65; *p* = 0.017). **Conclusions**: High-risk OSA is not more prevalent in adults with T1DM compared with controls; however, among individuals with T1DM, high-risk OSA is strongly and independently associated with microvascular complications. Given the potential contribution of diabetic microvascular and autonomic neuropathy to upper airway dysfunction, the relationship between OSA and vascular complications in T1DM may be bidirectional, warranting further longitudinal investigation.

## 1. Introduction

Obstructive sleep apnea (OSA) is a common sleep-related breathing disorder characterized by recurrent upper airway collapse during sleep, leading to intermittent hypoxemia, sleep fragmentation, and sympathetic activation [1,2]. Affecting approximately 9–38% of the general population, OSA is increasingly recognized as an important cardiometabolic risk factor through mechanisms including endothelial dysfunction, oxidative stress, systemic inflammation, and autonomic imbalance [3,4,5,6]. Despite its clinical significance, a substantial proportion of individuals with moderate-to-severe OSA remain undiagnosed [7].

Type 1 diabetes mellitus (T1DM) is a chronic autoimmune disease resulting in absolute insulin deficiency and affecting millions worldwide [3,4]. In addition to glycemic burden, individuals with T1DM frequently experience sleep disturbances, including poor sleep quality, nocturnal hypoglycemia, and sleep fragmentation, all of which may adversely affect metabolic control and daily functioning [8,9]. Emerging evidence suggests that sleep-disordered breathing may contribute to glycemic variability and diabetes-related complications [10,11,12].

While a strong bidirectional relationship between OSA and type 2 diabetes is well established, data in T1DM remain limited [13,14,15]. The pathophysiological interplay may differ, as T1DM is not primarily driven by obesity or insulin resistance. Nevertheless, OSA-related mechanisms—particularly intermittent hypoxia and sleep fragmentation—may promote microvascular injury through oxidative stress, inflammation, and endothelial dysfunction, processes directly implicated in diabetic retinopathy and neuropathy [16,17,18,19].

In addition, diabetic neuropathy, including autonomic dysfunction, may impair upper airway neuromuscular control, potentially increasing susceptibility to OSA. This suggests a bidirectional relationship in which OSA may contribute to microvascular complications, while advanced microvascular disease may in turn predispose to OSA [20,21,22,23].

Despite these biologically plausible links, the prevalence and clinical relevance of OSA in T1DM, particularly in relation to microvascular complications, remain insufficiently characterized. Moreover, commonly used OSA screening tools incorporate obesity and hypertension, which may confound risk assessment in T1DM populations. The modified Berlin Questionnaire (mBQ), which excludes these components, may provide a more appropriate assessment of OSA risk in this context [24,25,26,27].

Therefore, the present study aimed to compare the prevalence of high-risk OSA between adults with T1DM and controls and to investigate the association between high-risk OSA and microvascular complications among individuals with T1DM. We hypothesized that adults with T1DM classified as high risk for OSA would have a higher prevalence of diabetic microvascular complications than those at low risk.

## 2. Materials and Methods

### 2.1. Study Design and Participants

**Figure 1:**
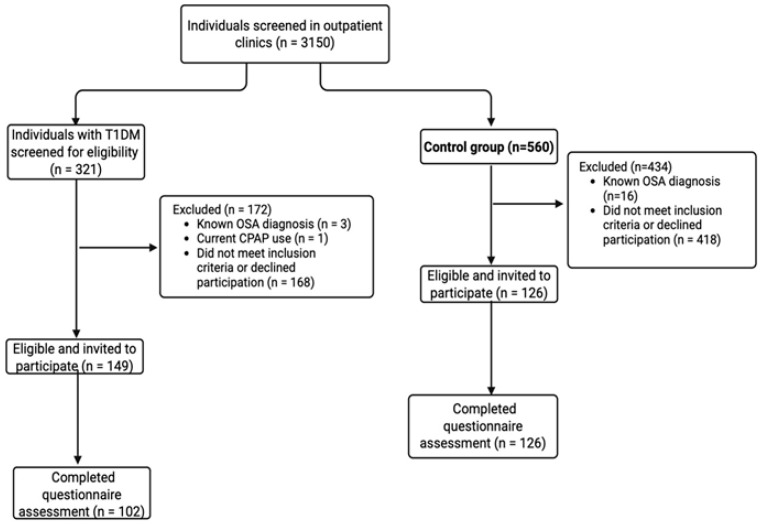
Flow diagram of participant selection for the T1DM and control groups. Abbreviations: CPAP, continuous positive airway pressure; OSA, obstructive sleep apnea; T1DM, type 1 diabetes mellitus.

This cross-sectional case–control study was conducted at the Department of Endocrinology and Metabolic Diseases, Koç University Hospital, between April 2023 and September 2024. Adults aged 18 years or older with a confirmed diagnosis of T1DM and control participants were eligible for inclusion (Figure 1). Participants with a prior diagnosis of OSA or current use of continuous positive airway pressure (CPAP) therapy were excluded from the study to avoid misclassification bias and potential modification of OSA-related risk. 

The diagnosis of T1DM was established based on clinical history and laboratory evidence, including low or undetectable C-peptide levels and/or the presence of pancreatic islet autoantibodies (anti-glutamic acid decarboxylase [GAD], insulinoma-associated antigen-2 [IA-2], or zinc transporter 8 [ZnT8] antibodies). Control participants were recruited from individuals attending routine outpatient evaluations at the same hospital and had no history of diabetes mellitus or major endocrine disorders. Controls were not formally matched to cases but were recruited from the same clinical population. To exclude the presence of undiagnosed diabetes, fasting and postprandial blood glucose levels were evaluated in all participants.

### 2.2. Data Collection and Clinical Variables

Data were collected using a structured online questionnaire (Qualtrics, Provo, UT) and electronic medical records. Demographic information included age and sex. Anthropometric data included height and weight obtained during outpatient clinic visit, from which body mass index (BMI) was calculated as kilograms divided by height in meters squared (kg/m^2^).

Clinical variables included smoking status (current smoker vs. former or non-smoker), alcohol use (yes/no), and physician-diagnosed hypertension. For participants with T1DM, diabetes duration, most recent glycated hemoglobin (HbA1c) levels, and the presence of diabetes-related microvascular and macrovascular complications were obtained from medical records. Among individuals with T1DM, data regarding insulin pump therapy, glucose monitoring frequency, and the modality of glucose monitoring (self-monitoring of blood glucose via finger-stick versus CGM) were documented. Clinical history of acute glycemic events—including self-reported episodes of hypoglycemia and hyperglycemia, as well as any related emergency department visits or hospital admissions—was collected through structured questionnaire items.

Diabetic retinopathy was defined based on documented ophthalmologic examination findings. Diabetic neuropathy included peripheral and/or autonomic neuropathy diagnosed according to standard clinical criteria and recorded in the medical charts. Diabetic neuropathy was evaluated using a combination of patient-reported symptoms and clinical examination findings. Diabetic peripheral neuropathy was diagnosed based on documented clinical symptoms and examination findings in medical records, including assessment using the 10-g Semmes–Weinstein monofilament test to evaluate loss of protective sensation. Diabetic nephropathy was evaluated based on the most recent estimated glomerular filtration rate (eGFR) and urinary albumin-to-creatinine ratio (UACR) obtained from medical records. Macrovascular complications included coronary artery disease, cerebrovascular disease, peripheral artery disease and diabetic foot.

### 2.3. Assessment of Obstructive Sleep Apnea Risk

Risk of OSA was assessed using the mBQ. The mBQ is a symptom-based screening tool derived from the original Berlin Questionnaire and excludes components related to obesity and hypertension, allowing assessment of OSA risk independently of these factors [26].

The questionnaire evaluates three symptom domains:

1. snoring characteristics (snoring intensity and frequency),
2. witnessed apneas during sleep, and
3. excessive daytime or morning sleepiness.

Each domain was considered positive if symptoms occurred at least three to four times per week or nearly every day. Participants were classified as having high-risk OSA if two or more symptom domains were positive; otherwise, they were classified as low risk.

### 2.4. Study Outcomes

The primary outcome of the study was the presence of microvascular complications, defined as diabetic retinopathy and/or diabetic neuropathy among participants with T1DM.

As secondary outcomes, individual components of microvascular complications were also evaluated separately. Macrovascular complications, including coronary heart disease, cerebrovascular disease, peripheral arterial disease, and diabetic foot, were recorded and are presented descriptively due to the limited number of events.

All analyses of diabetic complications were restricted to participants with T1DM, as these outcomes are not biologically applicable to controls.

### 2.5. Sample Size Considerations

Sample size estimation was based on detecting a medium effect size with 95% statistical power and a two-sided alpha of 0.05 for between-group comparisons. The final sample size exceeded the minimum number required for the primary analyses. A total of 102 individuals with T1DM and 126 controls were included.

### 2.6. Statistical Analysis

The Shapiro–Wilk test was used to assess the normality of continuous variables. Continuous variables are presented as mean ± standard deviation or median (interquartile range [IQR]), as appropriate. Categorical variables are presented as frequencies and percentages. Comparisons between groups were performed using the independent samples *t*-test or Mann–Whitney U test for continuous variables and the χ^2^ test or Fisher’s exact test for categorical variables, as appropriate.

The prevalence of high-risk obstructive sleep apnea (OSA) was compared between participants with T1DM and controls using χ^2^ tests. Analyses of microvascular complications were restricted to participants with T1DM.

To evaluate the association between high-risk OSA and microvascular complications (defined as the presence of diabetic retinopathy and/or neuropathy), multivariable logistic regression models were constructed. The primary model was adjusted for age and sex. In addition, a fully adjusted multivariable model was performed including age, sex, body mass index (BMI), hypertension, current smoking status, alcohol use, and glycated hemoglobin (HbA1c), to account for potential confounding.

As a secondary analysis, the composite vascular outcome was also evaluated descriptively; however, due to the limited number of macrovascular events, these findings were not used as the primary outcome in regression analyses.

Odds ratios (ORs) with 95% confidence intervals (CIs) were reported. All statistical analyses were performed using IBM SPSS Statistics for macOS (version 28.0; IBM Corp., Armonk, NY, USA). A two-sided *p* value < 0.05 was considered statistically significant. 

### 2.7. AI Statement

During the preparation of this work the authors used ChatGPT 5.2 in order to improve language and readability. After using this tool, the authors reviewed and edited the content as needed and take full responsibility for the content of the publication.

## 3. Results

### 3.1. Study Population and Baseline Characteristics

During the study period, 3150 individuals attending outpatient clinics were screened for eligibility (Figure 1). Among these, 321 individuals with T1DM and 560 healthy controls met the initial screening criteria. In the T1DM group, 172 individuals were excluded due to known OSA (n = 3), current CPAP use (n = 1), or failure to meet inclusion criteria or declining participation (n = 168). Consequently, 149 eligible individuals were invited to participate, of whom 102 completed the questionnaire and were included in the final analysis. In the control group, 434 individuals were excluded due to known OSA (n = 16) or failure to meet inclusion criteria or declining participation (n = 418), resulting in 126 controls who completed the questionnaire and were included in the analysis.

The mean age of the overall cohort was 38.8 ± 12.0 years. Individuals with T1DM were slightly older than controls (39.7 ± 10.5 vs. 38.0 ± 13.1 years, *p* = 0.045) and had a lower mean BMI (24.3 ± 3.8 vs. 25.5 ± 4.7 kg/m^2^, *p* = 0.033). Sex distribution did not differ significantly between groups.

Current smoking and alcohol use were more common among controls, whereas the prevalence of hypertension was similar between groups. Baseline characteristics of the study population are summarized in Table 1.

### 3.2. Prevalence of High-Risk OSA

Based on the mBQ, high-risk OSA was identified in 19 individuals with T1DM (18.6%) and in 15 controls (11.9%). The prevalence of high-risk OSA did not differ significantly between the T1DM and control groups (*p* = 0.156) (Table 2).

### 3.3. Microvascular Complications According to OSA Risk in T1DM

Analyses of microvascular complications were restricted to participants with T1DM. A composite microvascular outcome, defined as the presence of diabetic retinopathy and/or diabetic neuropathy, was identified in 28 individuals (27.5%). No cases of diabetic nephropathy were observed in the study cohort.

**Figure 2:**
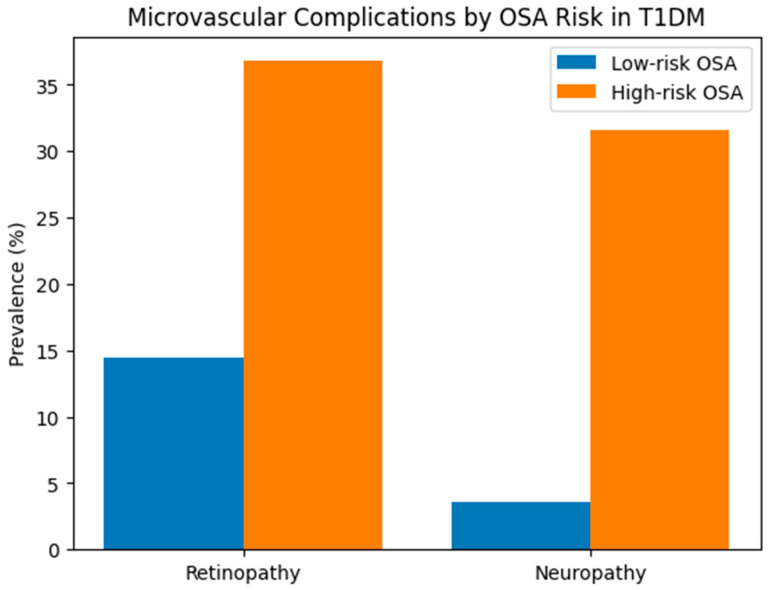
The number of microvascular complications in T1DM according to OSA Risk. Abbreviations: OSA, obstructive sleep apnea.

The prevalence of composite microvascular complications was significantly higher among individuals with high-risk OSA compared with those at low risk (68.4% vs. 18.0%, *p* < 0.001). Diabetic retinopathy and neuropathy were also more frequent in the high-risk OSA group when examined individually (Figure 2).

### 3.4. Descriptive Analysis of Macrovascular Complications According to OSA Risk in T1DM

Macrovascular complications were uncommon in this cohort. Coronary artery disease was identified in 4 patients, diabetic foot in 3 patients, peripheral artery disease in 1 patient, and cerebrovascular disease in 1 patient. Overall, 8.8% of participants had at least one macrovascular complication.

### 3.5. Association Between High-Risk OSA and Microvascular Complications in T1DM

In univariate logistic regression analysis, high-risk OSA was significantly associated with microvascular complications (OR 4.85; 95% CI 1.65–14.24; *p* = 0.004). Among clinical variables, hypertension (OR 4.20; 95% CI 1.32–13.36; *p* = 0.015) and HbA1c (OR 1.51; 95% CI 1.10–2.06; *p* = 0.011) were also significantly associated with microvascular complications. BMI showed a borderline association (OR 1.13; 95% CI 1.00–1.27; *p* = 0.056), whereas age, sex, smoking status, and alcohol use were not significantly associated (Table 3).

In the fully adjusted multivariable model including age, sex, body mass index, hypertension, smoking status, alcohol use, and HbA1c, high-risk OSA remained independently associated with microvascular complications (odds ratio [OR] 5.55; 95% confidence interval [CI] 1.36–22.65; *p* = 0.017) (Table 4).

## 4. Discussion

In this cross-sectional case–control study, we found that although the prevalence of high-risk OSA assessed by the mBQ was similar between adults with T1DM and controls, high-risk OSA was strongly associated with microvascular complications among individuals with T1DM. Notably, the strength of the association between high-risk OSA and microvascular complications increased after full adjustment, suggesting that this relationship is independent of traditional metabolic and cardiovascular risk factors, including glycemic control. In contrast, macrovascular events were infrequent in this relatively young cohort and should be interpreted descriptively and with caution. Importantly, the number of macrovascular events in our cohort was very limited, precluding any meaningful statistical inference. Therefore, our findings are restricted to microvascular complications, and the study is not powered to evaluate macrovascular outcomes.

A further noteworthy aspect of our findings is the differential strength observed across vascular phenotypes. In the present study, the primary outcome was defined as microvascular complications, specifically diabetic retinopathy and neuropathy. This definition reflects the structure of our data and avoids overinterpretation of composite outcomes. Microvascular complications in T1DM often precede overt macrovascular disease and may represent an earlier manifestation of cumulative vascular stress. In this context, OSA may be acting as an amplifier of microvascular injury rather than as a primary driver of established macrovascular pathology. The clustering of microvascular complications among individuals classified as high risk for OSA supports the hypothesis that sleep-disordered breathing may be particularly relevant in patients with longer disease duration and established vascular susceptibility.

Our findings suggest that OSA in T1DM may be clinically relevant not because it is more prevalent than in the general population, but because of its association with diabetes-related complications. The absence of a statistically significant difference in OSA risk between individuals with T1DM and controls may partly reflect the relatively young age and modest BMI of the study population. This contrasts with the well-established relationship between OSA and T2DM, in which OSA contributes to insulin resistance and poor glycemic control, whereas obesity, metabolic dysregulation, and elevated HbA1c increase the risk of OSA. In T1DM, where obesity and insulin resistance are not primary drivers, our results suggest that OSA may be linked to greater disease severity rather than a determinant of disease onset [28]. Recent evidence demonstrates a synergistic and potentially bidirectional relationship between obstructive sleep apnea (OSA) and diabetes-related vascular risk, indicating that OSA contributes to an increased cardiometabolic burden in individuals with diabetes. Large-scale observational cohort studies and narrative reviews have reported significant associations between OSA and higher prevalence of both microvascular and macrovascular complications, even in the absence of, or with minimal, traditional metabolic risk factors, as is frequently observed in T1DM subgroups. Collectively, these findings suggest that OSA may affect diabetes outcomes through mechanisms that extend beyond classic insulin resistance and glycemic dysregulation, which is consistent with the present findings in T1DM [29].

Several biological mechanisms may explain the observed association between OSA and microvascular complications in T1DM. Recurrent intermittent hypoxia and sleep fragmentation in OSA promote oxidative stress, systemic inflammation, endothelial dysfunction, and sympathetic activation, all of which play central roles in the development of diabetic angiopathy [28]. Collectively, these processes are central to diabetic microangiopathy.

Previous studies have linked OSA to increased prevalence and severity of diabetic retinopathy and neuropathy, independent of glycemic control and adiposity, although these data have been derived predominantly from T2DM populations [28,30]. In a cohort of 67 lean individuals with T1DM, Manin et al. demonstrated that longer diabetes duration was associated with a 4.54-fold increase in microvascular complications and an eightfold increase in macrovascular complications, underscoring the interplay between disease chronicity, vascular burden, and OSA risk [31]. In addition, at the molecular level, OSA-related hypoxia may impair nitric oxide bioavailability and microvascular autoregulation. Hypoxia-induced activation of pro-inflammatory cytokines and decreased nitric oxide bioavailability may impair microvascular autoregulation, capillary rarefaction, and increased vascular permeability. This mechanistic framework is increasingly supported by translational studies that identify OSA as a modulator of microvascular homeostasis, thereby elucidating how sleep-disordered breathing may exacerbate tissue ischemia and nerve fiber damage in individuals with diabetes [32,33]. To address potential confounding, we applied multivariable logistic regression models including age, sex, body mass index, hypertension, smoking status, alcohol use, and HbA1c. The association between high-risk OSA and microvascular complications remained significant after full adjustment, supporting the robustness of the findings. Notably, HbA1c was independently associated with microvascular complications, consistent with its established role in the pathogenesis of diabetic vascular damage.

While most mechanistic and observational research has concentrated on T2DM, recent studies indicate that greater severity of OSA is associated with an increased risk of diabetic peripheral nerve dysfunction, as evidenced by large cross-sectional analyses linking higher OSA severity to greater neuropathy burden. Additionally, the association between OSA and diabetic retinopathy is complex; some registries report a positive correlation, whereas others identify varying risk patterns. These varied clinical findings underscore the necessity for pathophysiologically focused research to elucidate the mechanisms by which sleep-disordered breathing and nocturnal hypoxemia contribute to microvascular tissue damage independent of glucose-mediated pathways [34].

Recent large-scale observational studies support a plausible association between OSA and microvascular complications, particularly affecting retinal and neural outcomes. For instance, real-world cohorts of patients with diabetic retinopathy have demonstrated higher rates of progression to vision-threatening complications and an increased need for ocular interventions among individuals with co-existing OSA. This finding aligns with the hypothesis that nocturnal hypoxemia may accelerate microvascular tissue injury [32]. Additionally, contemporary cross-sectional analyses have identified a positive correlation between OSA severity and the likelihood of diabetic peripheral neuropathy, indicating a graded relationship between sleep-disordered breathing and neural microvascular dysfunction. Although most available data are derived from type 2 diabetes or mixed diabetes populations, these findings collectively suggest that OSA-related hypoxic stress may contribute to microvascular disease progression in diabetes [35].

In T1DM, where traditional metabolic risk factors may be less influential, these results highlight the need to assess OSA risk, particularly in individuals with established microvascular complications.

Beyond a unidirectional model in which OSA contributes to microvascular injury, our findings also support the possibility of a bidirectional relationship between OSA and diabetic microvascular complications in T1DM. Diabetic autonomic and peripheral neuropathy may impair upper airway neuromuscular control, enhance central chemosensitivity to carbon dioxide, reduce pharyngeal dilator muscle responsiveness, and increase susceptibility to upper airway collapse during sleep [36,37]. Such mechanisms have been proposed in prior studies linking neuropathy to sleep-disordered breathing and may be particularly relevant in individuals with long-standing T1DM. Longer diabetes duration may also increase susceptibility to sleep-disordered breathing through mechanisms such as autonomic neuropathy and impaired ventilatory control. Although the cross-sectional design of our study precludes causal inference, the coexistence of OSA risk and microvascular complications raises the possibility of a self-perpetuating cycle in which OSA and microvascular disease exacerbate one another. Therefore, the present findings should be interpreted as associations rather than causal relationships, and the observed relationship between OSA and diabetic vascular complications may be bidirectional.

Given the established link between glycemic variability and vascular injury, strategies aimed at optimizing glycemic stability may also indirectly influence OSA-related vascular risk. In a study conducted among individuals with T2DM using CGM, glycemic variability was found to be significantly associated with the presence of diabetic neuropathy [38]. Expanding access to advanced diabetes technologies, including CGM and insulin pump therapy, may represent an important strategy to mitigate glycemic variability and reduce the burden of vascular complications in T1DM.

Importantly, the use of the mBQ in the present study is supported by our previously published validation study. In our prior validation study, the mBQ demonstrated acceptable diagnostic accuracy, for identifying individuals at high risk of OSA compared with polysomnography, while reducing confounding related to obesity and hypertension [26]. This is particularly relevant for populations such as individuals with T1DM, in whom traditional OSA risk factors may be less prominent. The strong and consistent association between high-risk OSA identified by the mBQ and microvascular complications observed in the present study further supports the clinical applicability of this screening tool in T1DM.

Several limitations should be acknowledged. First, the cross-sectional design precludes causal inference. Second, the relatively small sample size and low number of macrovascular events limit statistical power, particularly for macrovascular outcomes. Third, OSA was assessed using a screening questionnaire rather than polysomnography. Fourth, residual confounding cannot be entirely excluded despite multivariable adjustment. Fifth, the absence of diabetic nephropathy likely reflects the relatively young age of the cohort and moderate diabetes duration. Finally, selection bias cannot be excluded, as participation required completion of an online questionnaire. HbA1c and diabetes duration are clinically relevant factors in the development of diabetic complications and may also be associated with OSA risk; these variables should be examined further in larger longitudinal studies.

## 5. Conclusions

Our study demonstrates that high-risk OSA is not more prevalent in adults with T1DM than in controls; however, it is independently associated with microvascular complications, specifically diabetic retinopathy and neuropathy, among individuals with T1DM. These findings suggest that OSA risk may be clinically relevant in this population, particularly in patients with established microvascular disease.

However, these results should be interpreted with caution given the cross-sectional design and the limited sample size. In particular, the low number of macrovascular events precluded any meaningful conclusions regarding macrovascular outcomes.

Future large-scale, longitudinal studies incorporating objective sleep assessments are warranted to better clarify the temporal relationship and clinical implications of OSA in T1DM.

## Figures and Tables

**Table 1:** Baseline Characteristics of the Study Population.

Variable	Controls (n = 126)	T1DM (n = 102)	*p* Value
Age, years	38.0 ± 13.1	39.7 ± 10.5	0.045
Female sex, n (%)	68 (54.0)	56 (54.9)	0.888
BMI, kg/m^2^	25.5 ± 4.7	24.3 ± 3.8	0.033
Current smoker, n (%)	83 (65.9)	41 (40.2)	<0.001
Alcohol use, n (%)	37 (29.4)	17 (16.7)	0.025
Hypertension, n (%)	15 (11.9)	15 (14.7)	0.534
HbA1c (%)		7.0 (IQR 6.4–8.0)	
HbA1c ≥ 7%		55 (53.9)	
Microvascular complications			
Diabetic nephropathy		0 (0)	
Diabetic retinopathy		19 (18.6)	
Diabetic neuropathy		9 (8.8)	
Macrovascular complications			
Coronary artery disease		4 (3.9)	
Cerebrovascular disease		1 (1.0)	
Diabetic foot		3 (2.9)	
Peripheral artery disease		1 (1.0)	

Continuous variables are presented as mean ± standard deviation or median with interquartile ranges. Categorical variables are presented in percentages. *p* values were calculated using the independent samples *t* test for continuous variables and the χ^2^ test for categorical variables. Abbreviations: BMI, body mass index; HbA1c, hemoglobin A1c; T1DM, type 1 diabetes mellitus.

**Table 2:** Prevalence of High-Risk OSA Assessed by mBQ.

OSA Risk	Controls (n = 126)	T1DM (n = 102)	*p* Value
Low risk, n (%)	111 (88.1)	83 (81.4)	
High risk, n (%)	15 (11.9)	19 (18.6)	0.156

Abbreviations: OSA, obstructive sleep apnea; T1DM, type 1 diabetes mellitus.

**Table 3:** Univariate Variables Associated with Microvascular Complications in T1DM.

Variable	OR (95% CI)	*p* Value
High-risk OSA	4.85 (1.65–14.24)	0.004
Age	1.03 (0.99–1.08)	0.145
Sex (male)	1.02 (0.40–2.63)	0.970
BMI	1.13 (1.00–1.27)	0.056
Hypertension	4.20 (1.32–13.36)	0.015
Current smoking	1.67 (0.64–4.31)	0.292
Alcohol use	2.35 (0.76–7.31)	0.139
HbA1c	1.51 (1.10–2.06)	0.011

Abbreviations: BMI, body mass index; CI, confidence interval; HbA1c, glycated hemoglobin A1c; OR, odds ratio; OSA, obstructive sleep apnea; T1DM, type 1 diabetes mellitus.

**Table 4:** Multivariable Logistic Regression Analyses for Microvascular Complications in T1DM.

Model	Adjustments	OR for High-Risk OSA (95% CI)	*p* Value
Model 1	Age, sex	4.49 (1.37–14.70)	0.013
Model 2	+ BMI, hypertension, current smoking, alcohol use, HbA1c	5.55 (1.36–22.65)	0.017

Abbreviations: BMI, body mass index; CI, confidence interval; HbA1c, glycated hemoglobin A1c; OR, odds ratio; OSA, obstructive sleep apnea; T1DM, type 1 diabetes mellitus.

## Data Availability

Data collected for the study, including de-identified individual participant data, will be made available to others within 6 months after the publication of this article for academic purposes (e.g., meta-analyses), upon request to the corresponding author (yuksel.peker@lungall.gu.se), and with a signed data access agreement.

## References

[1] Gottlieb D.J., Punjabi N.M. (2020). Diagnosis and Management of Obstructive Sleep Apnea: A Review. JAMA.

[2] Chang J.L., Goldberg A.N., Alt J.A., Mohammed A., Ashbrook L., Auckley D., Ayappa I., Bakhtiar H., Barrera J.E., Bartley B.L. (2023). International Consensus Statement on Obstructive Sleep Apnea. Int. Forum Allergy Rhinol..

[3] Gregory G.A., Robinson T.I., Linklater S.E., Wang F., Colagiuri S., de Beaufort C., Donaghue K.C., Harding J.L., Wander P.L., Zhang X. (2022). Global incidence, prevalence, and mortality of type 1 diabetes in 2021 with projection to 2040: A modelling study. Lancet Diabetes Endocrinol..

[4] Atkinson M.A. (2012). The pathogenesis and natural history of type 1 diabetes. Cold Spring Harb. Perspect. Med..

[5] Lavie L. (2012). Oxidative stress inflammation and endothelial dysfunction in obstructive sleep apnea. Front. Biosci. (Elite Ed.).

[6] Dempsey J.A., Veasey S.C., Morgan B.J., O’Donnell C.P. (2010). Pathophysiology of sleep apnea. Physiol. Rev..

[7] American Diabetes Association Professional Practice Committee (2023). 2. Diagnosis and Classification of Diabetes: Standards of Care in Diabetes—2024. Diabetes Care.

[8] Villa M.P., Multari G., Montesano M., Pagani J., Cervoni M., Midulla F., Cerone E., Ronchetti R. (2000). Sleep apnoea in children with diabetes mellitus: Effect of glycaemic control. Diabetologia.

[9] Zhu B., Abu Irsheed G.M., Martyn-Nemeth P., Reutrakul S. (2021). Type 1 Diabetes, Sleep, and Hypoglycemia. Curr. Diab. Rep..

[10] Polonsky W.H., Fortmann A.L. (2022). Impact of Real-Time CGM Data Sharing on Quality of Life in the Caregivers of Adults and Children With Type 1 Diabetes. J. Diabetes Sci. Technol..

[11] Laffel L.M., Kanapka L.G., Beck R.W., Bergamo K., Clements M.A., Criego A., DeSalvo D.J., Goland R., Hood K., Liljenquist D. (2020). Effect of Continuous Glucose Monitoring on Glycemic Control in Adolescents and Young Adults With Type 1 Diabetes: A Randomized Clinical Trial. JAMA.

[12] Juvenile Diabetes Research Foundation Continuous Glucose Monitoring Study Group (2009). Factors predictive of use and of benefit from continuous glucose monitoring in type 1 diabetes. Diabetes Care.

[13] Lam J.C.M., Mak J.C.W., Ip M.S.M. (2012). Obesity, obstructive sleep apnoea and metabolic syndrome. Respirology.

[14] Janovsky C.C.P.S., Rolim L.C.d.S.P., de Sã¡ J.R., Poyares D., Tufik S., Silva A.B., Dib S.A. (2014). Cardiovascular autonomic neuropathy contributes to sleep apnea in young and lean type 1 diabetes mellitus patients. Front. Endocrinol..

[15] Reutrakul S., Thakkinstian A., Anothaisintawee T., Chontong S., Borel A.-L., Perfect M.M., Janovsky C.C.P.S., Kessler R., Schultes B., Harsch I.A. (2016). Sleep characteristics in type 1 diabetes and associations with glycemic control: Systematic review and meta-analysis. Sleep Med..

[16] Alshehri Z., Subramanian A., Adderley N.J., Gokhale K.M., Karamat M.A., Ray C.J., Kumar P., Nirantharakumar K., Tahrani A.A. (2022). Risk of incident obstructive sleep apnoea in patients with type 1 diabetes: A population-based retrospective cohort study. Diabetologia.

[17] Borel A., Benhamou P., Baguet J., Halimi S., Levy P., Mallion J., Pépin J. (2010). High prevalence of obstructive sleep apnoea syndrome in a Type 1 diabetic adult population: A pilot study. Diabet. Med..

[18] Atkeson A., Jelic S. (2008). Mechanisms of endothelial dysfunction in obstructive sleep apnea. Vasc. Health Risk Manag..

[19] Roy B. (2025). Pathophysiological Mechanisms of Diabetes-Induced Macrovascular and Microvascular Complications: The Role of Oxidative Stress. Med. Sci..

[20] Shields B.M., Peters J.L., Cooper C., Lowe J., Knight B.A., Powell R.J., Jones A., Hyde C.J., Hattersley A.T. (2015). Can clinical features be used to differentiate type 1 from type 2 diabetes? A systematic review of the literature. BMJ Open.

[21] Pop-Busui R., Boulton A.J., Feldman E.L., Bril V., Freeman R., Malik R.A., Sosenko J.M., Ziegler D. (2017). Diabetic Neuropathy: A Position Statement by the American Diabetes Association. Diabetes Care.

[22] Callaghan B.C., Kerber K.A., Lisabeth L.L., Morgenstern L.B., Longoria R., Rodgers A., Longwell P., Feldman E.L. (2014). Role of neurologists and diagnostic tests on the management of distal symmetric polyneuropathy. JAMA Neurol..

[23] Tahrani A.A., Ali A., Raymond N.T., Begum S., Dubb K., Mughal S., Jose B., Piya M.K., Barnett A.H., Stevens M.J. (2012). Obstructive sleep apnea and diabetic neuropathy: A novel association in patients with type 2 diabetes. Am. J. Respir. Crit. Care Med..

[24] Abbasi A., Gupta S.S., Sabharwal N., Meghrajani V., Sharma S., Kamholz S., Kupfer Y. (2021). A comprehensive review of obstructive sleep apnea. Sleep Sci..

[25] Abrishami A., Khajehdehi A., Chung F. (2010). A systematic review of screening questionnaires for obstructive sleep apnea. Can. J. Anaesth..

[26] Celik Y., Baygül A., Peker Y. (2023). Validation of the Modified Berlin Questionnaire for the Diagnosis of Obstructive Sleep Apnea in Patients with a History of COVID-19 Infection. J. Clin. Med..

[27] Chou K., Chang Y., Chen Y., Su K., Perng D., Chang S., Shiao G. (2011). The minimum period of polysomnography required to confirm a diagnosis of severe obstructive sleep apnoea. Respirology.

[28] Passali D., Bellussi L.M., Santantonio M., Passali G.C. (2025). A Structured Narrative Review of the OSA–T2DM Axis. J. Clin. Med..

[29] Valensi P., Benmohammed K., Zerguine M. (2025). Bidirectional interplay of sleep apnea syndrome and cardio-vascular disorders in diabetes. Diabetes Res. Clin. Pract..

[30] Gentile S., Monda V.M., Guarino G., Satta E., Chiarello M., Caccavale G., Mattera E., Marfella R., Strollo F. (2025). Obstructive Sleep Apnea and Type 2 Diabetes: An Update. J. Clin. Med..

[31] Manin G., Pons A., Baltzinger P., Moreau F., Iamandi C., Wilhelm J.M., Lenoble P., Kessler L., Kessler R. (2015). Obstructive sleep apnoea in people with Type 1 diabetes: Prevalence and association with micro- and macrovascular complications. Diabet. Med..

[32] Rahimy E., Koo E.B., Wai K.M., Ludwig C.A., Kossler A.L., Mruthyunjaya P. (2025). Impact of Obstructive Sleep Apnea on Diabetic Retinopathy Progression and Systemic Complications. Arch. Ophthalmol..

[33] Zhao L., Liu Z., Zhang R., Li Y., Zhang X. (2025). Causal relationship between obstructive sleep apnea and diabetic nephropathy: Bidirectional and multivariable Mendelian randomization study. Ren. Fail..

[34] Lu N., Cheng G., Qian Y., An D., Yin F., Hou Y., Liu X., Lu Q., Ma C., Wang R. (2025). The association between obstructive sleep apnoea and diabetic peripheral neuropathy in subjects with type 2 diabetes. Front. Endocrinol..

[35] Eleftheriadou A., Spallone V., Tahrani A.A., Alam U. (2024). Cardiovascular autonomic neuropathy in diabetes: An update with a focus on management. Diabetologia.

[36] Bottini P., Scionti L., Santeusanio F., Casucci G., Tantucci C. (2000). Impairment of the respiratory system in diabetic autonomic neuropathy. Diabetes Nutr. Metab..

[37] Tantucci C., Scionti L., Bottini P., Dottorini M.L., Puxeddu E., Casucci G., Sorbini C.A. (1997). Influence of autonomic neuropathy of different severities on the hypercapnic drive to breathing in diabetic patients. Chest.

[38] Kalopita S., Liatis S., Thomakos P., Vlahodimitris I., Stathi C., Katsilambros N., Tentolouris N., Makrilakis K. (2014). Relationship between autonomic nervous system function and continuous interstitial glucose measurement in patients with type 2 diabetes. J. Diabetes Res..

